# Integrating protein and DNA embeddings for improving genome-wide transcription factor binding site prediction

**DOI:** 10.1093/nargab/lqag047

**Published:** 2026-05-06

**Authors:** Shreya Basnet, Jianlin Cheng

**Affiliations:** Department of Electrical Engineering & Computer Science, University of Missouri, Columbia, MO 65211, United States; NextGen Precision Health, University of Missouri, Columbia, MO 65211, United States; Department of Electrical Engineering & Computer Science, University of Missouri, Columbia, MO 65211, United States; NextGen Precision Health, University of Missouri, Columbia, MO 65211, United States

## Abstract

Transcription factors (TFs) regulate gene expression by binding to specific DNA sites on genome, making accurate TF binding site prediction critical for understanding gene regulation and downstream phenotypes. Almost all current deep learning methods use only DNA-related information to predict TF binding sites, ignoring the fact that different TF protein sequences and structures recognize distinct DNA patterns. Not leveraging TF information not only limits prediction accuracy but also makes the methods not generalizable to predicting binding sites of new TFs that do not exist in the training data. Here, we present TransBind, a protein-aware deep learning architecture that integrates DNA sequence information with protein embeddings containing both sequence and structural information derived from a protein language model pretrained on DNA-binding proteins, to improve TF binding site prediction. Through the cross-attention, a TF embedding selectively attends to genomic regions according to its unique binding properties. Evaluated on the data of 690 ChIP-seq experiments spanning 161 TFs across 91 human cell types, TransBind achieves an AUROC of 0.9508 and AUPR of 0.3741—representing a $\ge$11.8% relative AUPR improvement over state-of-the-art methods including TBiNet, EPBDXDNABERT-2, DanQ, and DeepSEA. The model outperformed existing methods in $\ge$98% of TF–cell type combinations. It also recovered 160 known TF binding motifs in the JASPAR database, providing the biological interpretability of the model. Moreover, the approach enables label-zero-shot prediction for unseen TFs, demonstrating its potential of generalizing to new, poorly characterized TFs. The source code of TransBind is available at https://github.com/jianlin-cheng/TransBind. The version used in this work is archived at https://doi.org/10.5281/zenodo.19462292.

## Graphical Abstract

**Graphical Abstract:**
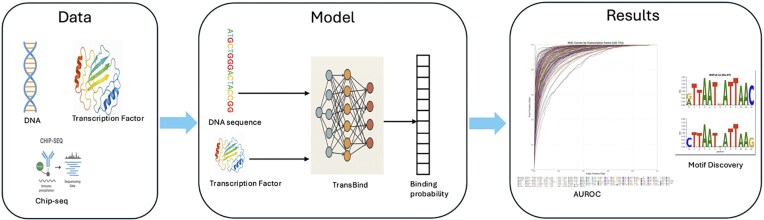

## Introduction

Transcription factors (TFs) are proteins that bind specific DNA sequences to regulate a broad range of cellular processes in almost all species. By activating or repressing target genes, TFs form the backbone of gene regulatory networks, controlling gene expression and protein abundance [1]. For instance, there are over 1600 predicted TFs in the human genome interacting with millions of potential binding sites [2]. Mapping the TF–DNA interaction is critical for understanding gene regulation and for linking phenotypic outcomes, such as diseases, to variants in noncoding regulatory regions that disrupt TF binding and alter transcriptional programs [2, 3].

Chromatin immunoprecipitation followed by sequencing (ChIP-seq) [4] is the gold standard for identifying genome-wide TF binding sites, but its scalability is limited by high sequencing costs, substantial material requirements, and dependence on high-quality TF-specific antibodies. These constraints have driven the development of computational approaches to predict TF–DNA binding directly from genomic sequence.

Early models, including position weight matrices [5], hidden Markov models [6, 7], and support vector machines [8, 9] could capture DNA sequence preferences of TFs but had a high false-positive rate and relied heavily on hand-crafted features.

Deep learning methods transformed the field by enabling end-to-end learning from raw DNA sequences. Convolutional neural networks (CNNs), such as DeepBind [10], learned sequence motifs directly, while multitask models such as DeepSEA [11] jointly predicted hundreds of chromatin features, improving performance through shared representations. Hybrid CNN–RNN architectures like DanQ [12] captured long-range dependencies, and attention-based methods such as TBiNet [13] and DeepGRN [14] improved interpretability by focusing on the most informative sequence regions.

Despite these advances, DNA sequence-only models neglect important biophysical determinants of TF binding. A growing body of work has demonstrated that DNA structural properties,including minor groove width (MGW), roll, propeller twist (ProT), and helix twist (HelT), play a critical role in protein–DNA recognition. These DNA shape features, originally derived from Monte Carlo simulations [8, 15, 16], have been shown to improve binding specificity when combined with sequence information. Beyond static structure, DNA breathing dynamics computed using the nonlinear Peyrard–Bishop–Dauxois (PBD) model [17] have been applied to regulatory prediction tasks such as transcription start site identification [18]. Frameworks such as DLBSS [19] integrated DNA shape features with deep convolutional networks for protein-binding microarray data, while DNAffinity [20] incorporated structural and mechanical properties derived from atomistic molecular dynamics simulations. More recently, advances in DNA/genome foundation models pretrained on large genomic corpora have provided a powerful paradigm for capturing sequence context across multiple scales. DNABERT-2 [21], a transformer-based DNA language model, employs byte pair encoding to tokenize sequences into variable-length subsequences and uses self-attention to encode both local nucleotide patterns and long-range dependencies. These representations significantly improve performance on tasks such as TF binding site prediction and variant effect analysis. Building on these advances, recent hybrid approaches have begun to integrate foundation models with biophysical constraints. EPBD×DNABERT-2 [22] combines DNABERT-2 embeddings with features derived from the extended PBD model to capture sequence-dependent breathing dynamics and thermodynamic stability of the DNA double helix. By integrating learned sequence representations with physical constraints on DNA structure and flexibility, this approach produces expressive and interpretable embeddings that enhance TF–DNA binding prediction.

Despite this progress, nearly all existing methods rely exclusively on DNA sequence and related genomic features, treating TFs as interchangeable and ignoring critical determinants of binding specificity such as the TF amino acid sequence and three-dimensional structure [23, 24]. A recent study, BTFBS [25], was the first to incorporate TF protein sequences into binding prediction, but it relied on small curated binding-site databases rather than genome-wide data. Moreover, TFs were represented only as raw amino acid tokens processed by convolutional networks, without leveraging contextual or evolutionary protein embeddings. The lack of effective TF modeling limits predictive accuracy and generalization, particularly for TFs with sparse binding data, and precludes label-zero-shot prediction for unseen TFs.

To address these limitations, it is essential to integrate both DNA and TF information in a unified framework. Protein language models (PLMs) offer a powerful means of representing TFs, learning rich contextual embeddings from millions of protein sequences that capture structural and functional properties. Models such as ESM-2 [26] provide general-purpose protein representations, while domain-adapted variants such as ESM–DBP [27] specialize in DNA-binding proteins and encode biochemical features directly relevant to TF–DNA recognition.

Here, we present TransBind, a protein-aware deep learning architecture that integrates DNA sequence encodings with TF protein embeddings generated by PLMs via a cross-attention mechanism to predict TF–DNA binding sites. This design allows each TF to selectively attend to genomic regions based on its unique binding properties. Evaluated on a large-scale dataset, TransBind significantly outperforms state-of-the-art deep learning methods and supports label-zero-shot prediction for TFs unseen during training. By uniting protein and DNA features, TransBind advances both the accuracy and biological interpretability of TF–DNA binding prediction.

## Materials and methods

### DNA data

To train and evaluate TF–DNA binding prediction, we used EPBDXDNA dataset [22], derived from ENCODE (encyclopedia of DNA elements) ChIP-seq experiments. EPBDXDNA includes 690 TF-cell type experiments spanning 161 TFs across 91 human cell types. Multiple experiments per TF in different cell types capture diverse cellular contexts and experimental conditions. The EPBDXDNA dataset is the updated version of DeepSEA dataset [11] based on the recent genome assembly. The TF-binding peaks were called by the standardized processing pipeline provided by the ENCODE analysis working group.

Based on the GRCh37 (hg19) reference genome [28], we partitioned the genome into nonoverlapping 200 bp bins. A bin was labeled positive if at least 50% of its length overlapped a ChIP-seq peak [11]; otherwise, it was labeled negative. To provide a sufficient context for TF binding site prediction, each bin was extended by 400 bp on either side, resulting in a 1000 bp input DNA sequence.

In cases where multiple peaks from the same experiment overlapped a single bin, we resolved duplicates by retaining the first instance and merging their labels. After de-duplication, we obtained 1 903 712 unique labeled bins. Removing sequences containing ambiguous nucleotides (“N”) yielded a final set of 1 903 668 bins, covering ~12.6% of the genome. Reverse complement sequences of the bins were also incorporated into the training, validation, and test sets, effectively doubling the total number of samples to 3,807,336.

Each 1000 bp genomic sequence bin was one-hot encoded as a $1000 \times 4$ matrix representing the nucleotides A, C, G, and T. For each of the 690 TF-cell type experiments, a binary value was assigned to each bin indicating whether it has a TF binding site in the experiment or not (positive or negative). This resulted in a binary label vector of length 690 for each bin, indicating in which TF-cell type experiments the bin is positive or negative.

To ensure consistency with prior work, we adopted the chromosome-based data split used in DeepSEA [11]. The bins of chromosomes 8 and 9 were held out for testing, the bins of chromosome 7 were used for validation, and the bins of the remaining autosomes plus chromosome X (excluding Y) were used for training. The resulting data distribution is summarized in Table 1.

**Table 1.** Chromosome-based data partitioning used for training, validation, and testing

Split	Chromosome(s)	Bins	%
Train	1–22, X (excl. 7–9)	3 272 910	85.96
Validation	7	203 784	5.35
Test	8, 9	330 642	8.69
**Total**		3 807 336	100.00

To verify that the chromosome-based data split prevents sequence leakage between partitions, we computed pairwise 8-mer Jaccard similarity between 4000 randomly sampled DNA sequences from each dataset split. The cross-split similarities were uniformly low (training-validation: mean 0.0138 ± 0.0071, training-test: mean 0.0136 ± 0.0067, validation-test: mean 0.0137 ± 0.0069). The median similarities are also consistent across comparisons ( 0.0127–0.0128). These results indicate minimal sequence overlap between partitions, suggesting that the chromosome-based splitting strategy effectively prevents DNA sequence leakage.

**Figure 1.**
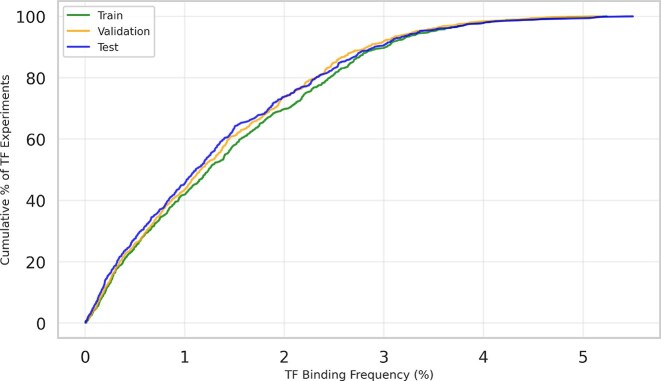
Cumulative distribution of TF binding frequencies in the training, validation, and test sets, respectively. The plot shows the percentage of the TF-cell type experiments (*y*-axis) with binding frequencies at or below each threshold (*x*-axis), demonstrating that most TFs bind to a very low proportion of genomic regions while only a small fraction of TFs exhibit substantial binding activity ($> 3\%$) in each set.

Figure 1 illustrates the distribution of TF binding frequencies across the 690 ChIP-seq TF-cell type experiments in the training, validation, and test sets, respectively. The cumulative distribution graph demonstrates severe class imbalance in TF binding frequencies: in approximately 50% of the TF-cell type experiments the TF binds to fewer than 1% of bins, while in 75% it binds to fewer than 2% of bins. Only a small fraction of TFs exhibit binding activity above 3% frequency. This pronounced skew toward low binding frequencies is consistent across all three dataset splits (train, validation, and test), confirming that positive binding sites are very rare. With positive examples comprising only 1.44% bins across 690 TF-cell type experiments, the dataset is therefore highly imbalanced, with the vast majority of examples being negative.

### Transcription factor data

The amino acid sequences of the 161 TFs were retrieved from UniProt. To evaluate sequence diversity in our training set, we performed pairwise sequence similarity analysis using global sequence alignment across all 161 TFs. The analysis revealed substantial diversity, with a mean pairwise identity of 19.13% (median: 20.00%, range: 3.77%–77.33%). Only one TF pair (HDAC1 and HDAC2, 77.33% identity) exceeded the 70% similarity threshold, representing $< 0.01\%$ of all comparisons. This low sequence redundancy indicates that the training set spans diverse TF families and supports learning TF-specific binding preferences rather than relying on sequence similarity.

We used ESM–DBP, a domain-adapted PLM trained specifically for DNA-binding proteins [27], to generate embeddings for each TF. ESM–DBP extends the general-purpose PLM ESM2 [26] by fine-tuning the pretrained ESM2 on 170,264 non-redundant DNA-binding protein sequences from UniProtKB [29], thereby incorporating TF-specific knowledge. Each TF sequence was processed with ESM–DBP to produce an embedding of shape $L \times d_2$, where $L$ is the sequence length and $d_2$ is the embedding dimension. For TF–DNA binding site prediction, a single $d_2$-dimensional feature vector for each TF was obtained by averaging the embedding across the sequence length.

### Deep learning model for TF–DNA binding site classification

**Figure 2.**
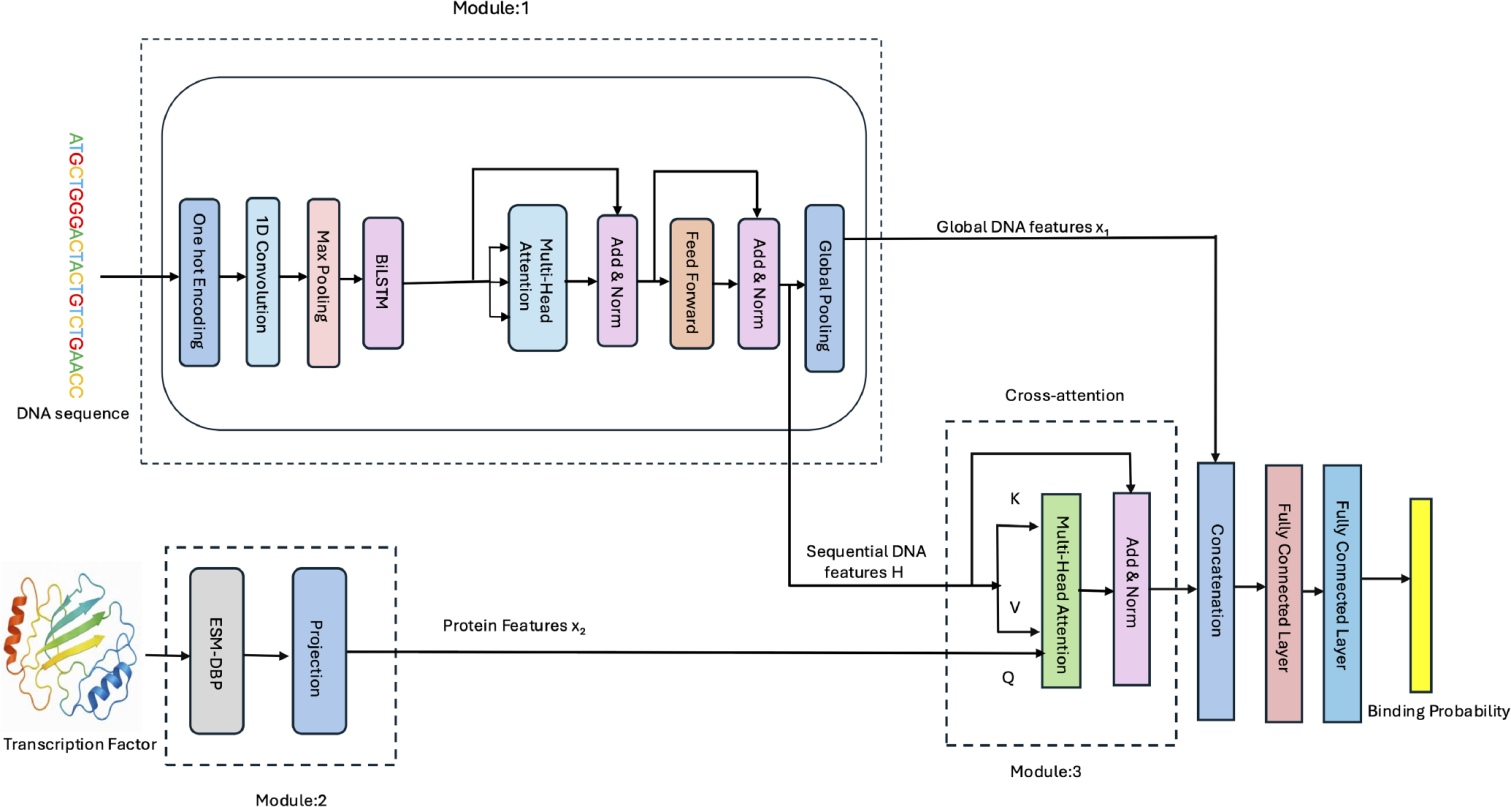
Overview of the TransBind architecture. Module 1 encodes the DNA sequence of a bin using convolutional, BiLSTM, and transformer layers to generate a contextual DNA representation (embedding). Module 2 encodes TF sequences using pretrained ESM–DBP embeddings and linear projection. Module 3 applies the cross-attention between TF and DNA features to predict TF–DNA binding probability.

All the existing deep learning methods were trained to predict binding sites of a predetermined set of TFs (or TF-cell type combinations) with the availability of some experimentally determined DNA binding sites that can be used for training. The problem is often formulated as a multilabel classification problem, i.e. predicting if a DNA bin contains a binding site of each TF in the set. It is treated as multilabel classification because one bin may be the binding site of multiple TFs. Following this paradigm, we designed the architecture of TransBind to integrate protein and DNA information to predict the binding sites of a set of TF experiments (i.e. 690 TF-cell type experiments in this study) with labeled training data (Fig. 2). It has three modules: (i) DNA sequence encoder, (ii) protein encoder, and (iii) bimodal feature aggregation for TF–DNA binding prediction, which are described in detail below.

#### Module 1: DNA sequence encoder

The binary matrix of shape $1000 \times 4$ encoding the DNA sequence of each bin is first transposed to the shape $4 \times 1000$ to match the input format of the following convolutional layer (see Module 1 in Fig. 2). The transposed matrix is passed through a one-dimensional convolutional layer with 320 filters (kernels) and a kernel size of 26, designed to capture local sequence motif patterns. The convolutional output is followed by a ReLU activation function and a one-dimensional max-pooling operation with a window size of 13, reducing the length of generated hidden features while retaining the strongest activations. These operations progressively reduce the sequence length from the original 1000 bp input to an effective sequence length of L = 75 positions.

The pooled features are then entered into a two-layer bidirectional long- and short-term memory block (BiLSTM) with a hidden layer of 160 hidden nodes in each direction, resulting in a $d_1$-dimensional contextualized representation at each sequence position ($d_1=320$). This enables the model to capture long-range dependencies between features from both upstream and downstream sequence contexts.

The transformer encoder has a multihead self-attention layer with 16 attention heads and a feed-forward layer with 1024 hidden nodes, allowing the model to attend informative features globally.

Finally, the DNA encoder produces two representations: (i) $\mathbf {H} \in \mathbb {R}^{L \times d_1}$, the position-wise features from the transformer encoder (with $L=75$ and $d_1=320$), which preserve spatial information along the sequence; and (ii) $\mathbf {x}_1 \in \mathbb {R}^{d_1}$, a global embedding obtained by applying average pooling to $\mathbf {H}$, serving as a compact summary of the sequence.

$\mathbf {H}$ is integrated with the protein embeddings generated in Module 2 through the cross-attention in Module 3, while $\mathbf {x}_1$ is concatenated with the output of the cross-attention to predict TF–DNA binding in Module 3.

#### Module 2: Protein encoder

TransBind leverages ESM–DBP, a domain-adapted variant of ESM-2 fine-tuned for DNA-binding proteins, to generate the embedding for each TF, which can capture sequence and structural features relevant to DNA–protein interactions (see Module 2 in Fig. 2). For each of the 161 unique TFs in the dataset, a fixed-length embedding of dimension $d_2$ ($d_2$ = 1280) is generated by ESM–DBP. This fixed-length embedding is achieved by mean pooling over the per-residue embeddings generated by ESM–DBP.

To facilitate the integration with the DNA sequence embeddings from Module 1, each protein embedding is projected into a $d_1$-dimensional latent space using a learnable linear transformation:


(1) \begin{eqnarray*}
\mathbf {x}_2 = \mathbf {W}_p \mathbf {e}_p + \mathbf {b}_p, \quad \mathbf {x}_2 \in \mathbb {R}^{d_1} ,
\end{eqnarray*}

where $\mathbf {e}_p \in \mathbb {R}^{d_2}$ is the original ESM–DBP protein embedding, $\mathbf {W}_p \in \mathbb {R}^{d_1 \times d_2}$ is a trainable projection matrix, and $\mathbf {b}_p \in \mathbb {R}^{d_1}$ is a bias term. The resulting protein vector $\mathbf {x}_2$ has the same dimensionality ($d_1$) as the DNA embedding to facilitate the subsequent cross-modal interaction in Module 3.

#### Module 3: Bimodal feature aggregation for TF–DNA binding prediction

A key innovation of TransBind lies in a cross-attention mechanism that enables bimodal interaction between DNA and TF embeddings (features) (see Module 3 in Fig. 2). Biologically, each TF “scans” the DNA sequence to focus on relevant binding regions. We implement this using cross-attention, where the TF embedding acts as the query, while the contextualized DNA embeddings serve as keys and values.

Formally, let $\mathbf {H} \in \mathbb {R}^{L \times d_1}$ denote the contextualized DNA embeddings from Module 1, where $L$ is the effective sequence length after convolution and pooling, and let $\mathbf {x}_2 \in \mathbb {R}^{d_1}$ be the projected TF embedding from Module 2. The cross-attention is computed as:


(2) \begin{eqnarray*}
\mathrm{Attention}(\mathbf {q}, \mathbf {K}, \mathbf {V}) = \mathrm{softmax}\left( \frac{\mathbf {q} \mathbf {K}^\top }{\sqrt{d}} \right) \mathbf {V},
\end{eqnarray*}

where $\mathbf {q} = \mathbf {x}_2 \mathbf {W}_q \in \mathbb {R}^{1 \times d}$ is the TF query vector, and $\mathbf {K} = \mathbf {H} \mathbf {W}_k \in \mathbb {R}^{L \times d}$ and $\mathbf {V} = \mathbf {H} \mathbf {W}_v \in \mathbb {R}^{L \times d}$ are the key and value matrices derived from the DNA embeddings. Here, $\mathbf {W}_q, \mathbf {W}_k, \mathbf {W}_v \in \mathbb {R}^{d_1 \times d}$ are learnable projection matrices, and $d$ is the attention dimensionality.

All 690 TF protein embeddings are processed in parallel in a single multihead cross-attention operation. Each TF acts as a separate query, producing TF-specific attended DNA representations simultaneously. These representations are aggregated via mean pooling into a single TF-aware summary vector $\mathbf {z} \in \mathbb {R}^{d}$, which captures DNA binding information conditioned on the set of TFs.

TF information enters the model through the protein embeddings used as attention queries, while cell-type specificity is captured implicitly through label-specific classifier weights rather than explicit cell-type inputs. This design ensures that cross-attention is computed efficiently once per sample while enabling large-scale TF–cell type prediction.

### Training and evaluation

TransBind was implemented using PyTorch Lightning. It was trained on the training data and its hyperparameter optimization was performed on the validation data using Optuna (see Supplementary Note S1 for details).We additionally performed a hyperparameter sensitivity analysis across all Optuna trials using Spearman rank correlation to assess the robustness of the selected hyperparameters (Supplementary Fig. S1). The final optimized model employed a 1D CNN (320 channels, kernel size 26), 2-layer bidirectional LSTM (160 hidden units), and transformer encoder with 16 attention heads. The cross-attention mechanism also has 16 heads. Training utilized binary cross-entropy with logits loss, the AdamW optimizer (learning rate $3.28 \times 10^{-4}$, weight decay 0.028) with cosine annealing scheduling over 60 epochs, dropout regularization ($P=0.088$), and batch size 256.

The performance of TransBind and baseline methods was evaluated using AUROC (area under the receiver operating characteristic curve) and AUPR (area under the precision–recall curve). The task is formulated as a multilabel classification problem, where each label corresponds to a specific TF–cell type pair, resulting in a total of 690 labels. For each label, predictions across all test sequences were compared to the corresponding binary ground-truth labels to compute per-label AUROC and AUPR scores. These scores were then macro-averaged across all labels to obtain the overall metrics reported in Table 2, ensuring equal contribution from each TF–cell type experiment. AUROC measures the model’s ability to rank positive samples higher than negatives irrespective of class imbalance, while AUPR is particularly informative for sparse TF binding data by capturing the precision–recall trade-off.

**Table 2.** AUROC and AUPR of TransBind and other methods on the test dataset

Method	AUROC	AUPR
DeepSEA	0.8934	0.2509
DanQ	0.9254	0.3065
TBiNet	0.9402	0.3346
finetuned DNABERT-2	0.9180	0.2960
EPBDXDNABERT-2	0.9490	0.3260
**TransBind**	**0.9508**	**0.3741**

### Deep learning model for label-zero-shot TF–DNA binding site prediction

To enable prediction of DNA binding sites for TFs unseen during training, we reformulate TF–DNA binding prediction from a multilabel classification problem in the previous section into a general binary classification task. Instead of simultaneously predicting binding probabilities for a preselected set of TFs given a DNA bin, the new model addresses a fundamental question: given an arbitrary single TF and a DNA bin, will they bind? Except for the difference in the final output layer in Module 3, the new model (TransBind_zeroshot) is the same as TransBind for multilabel classification. Rather than learning binding patterns of a predetermined set of TFs, TransBind_zeroshot is trained to capture the universal principles of any protein–DNA interactions that underlie binding affinity and specificity.

One key architectural insight is that the cross-attention mechanism in TransBind_zeroshot operates as a shared module for processing individual TF–DNA pairs. During training, each DNA sequence is paired with multiple TF embeddings and vice versa, enabling the model to learn sequence motifs and structural determinants of compatibility. This setup compels the model to extract generalizable features of protein–DNA recognition—such as amino acid–nucleotide contact preferences, structural complementarity, and physicochemical compatibility.

To further promote generalization, we applied regularization strategies in training: randomly masking entire TF embeddings with probability 0.05 and injecting small Gaussian noise ($\sigma = 0.01$) into protein representations. These techniques discourage reliance on specific TF identities and improve robustness to variability in protein features.

Consequently, once trained, the model can predict binding for entirely novel TFs by simply providing their ESM–DBP embedding together with a DNA sequence, without requiring additional training or fine-tuning. This label-zero-shot prediction capability substantially improves the model’s flexibility and applicability, offering a general framework for TF–DNA binding prediction across arbitrary TFs.

For training and validation, we used the same dataset as TransBind. The test of label-zero-shot prediction was blindly carried out on the data of three TFs (HNF1A, ATF4, and FOXA3) excluded from training and validation. This setup ensured the model was tested on truly unseen TFs, directly assessing its ability to generalize to novel TF–DNA interactions.

## Results

### Comparison with state-of-the-art TF–DNA binding prediction methods

To rigorously evaluate TransBind, we adopted the same benchmarking framework used in previous state-of-the-art methods, including DeepSEA [11], DanQ [12], DNABERT-2 [21], and TBiNet [13], and compared all models on the same test dataset. For fair comparison, all baseline models (DeepSEA, DanQ, TBiNet, and DNABERT-2) were retrained on our training dataset using the same Optuna-based hyperparameter optimization protocol as TransBind, including identical search spaces, numbers of trials, early-stopping criteria, and random seeds. In all cases, the optimized configurations achieved performance lower than the originally reported results, and the original setting was used for evaluation. The final hyperparameter configurations for each retrained baseline are summarized in Supplementary Table S1. The EPBDXDNABERT-2 model could not be retrained due to unavailable training code and data; we therefore report its performance as described in the original publication. We report area under the AUROC and AUPR. AUROC measures a model’s ability to distinguish between positive and negative classes across varying thresholds, while AUPR emphasizes performance on the positive class, making it particularly informative for highly imbalanced TF–DNA binding datasets.

As shown in Table 2, TransBind outperforms all baseline models, achieving an AUROC of 0.9508 and an AUPR of 0.3741. In terms of AUPR, compared to the second-best model TBiNet, it has an absolute improvement of 0.0412. For AUROC, compared to the second-best model EPBDXDNABERT-2 (0.9490), it yields an absolute improvement of 0.0125; since the underlying predictions for EPBDXDNABERT-2 were not available, we report only the absolute difference here. Using the available TBiNet predictions, we performed paired *t*-tests comparing TransBind against TBiNet across all 690 TF–cell type combinations. TransBind shows highly significant improvements in both AUROC (*P* = $1.75 \times 10^{-93}$, Cohen’s *d* = 0.92) and AUPR (*P* = $3.84 \times 10^{-80}$, Cohen’s *d* = 0.83). The highly significant difference indicates that the observed improvements are both statistically robust and practically meaningful.

**Figure 3.**
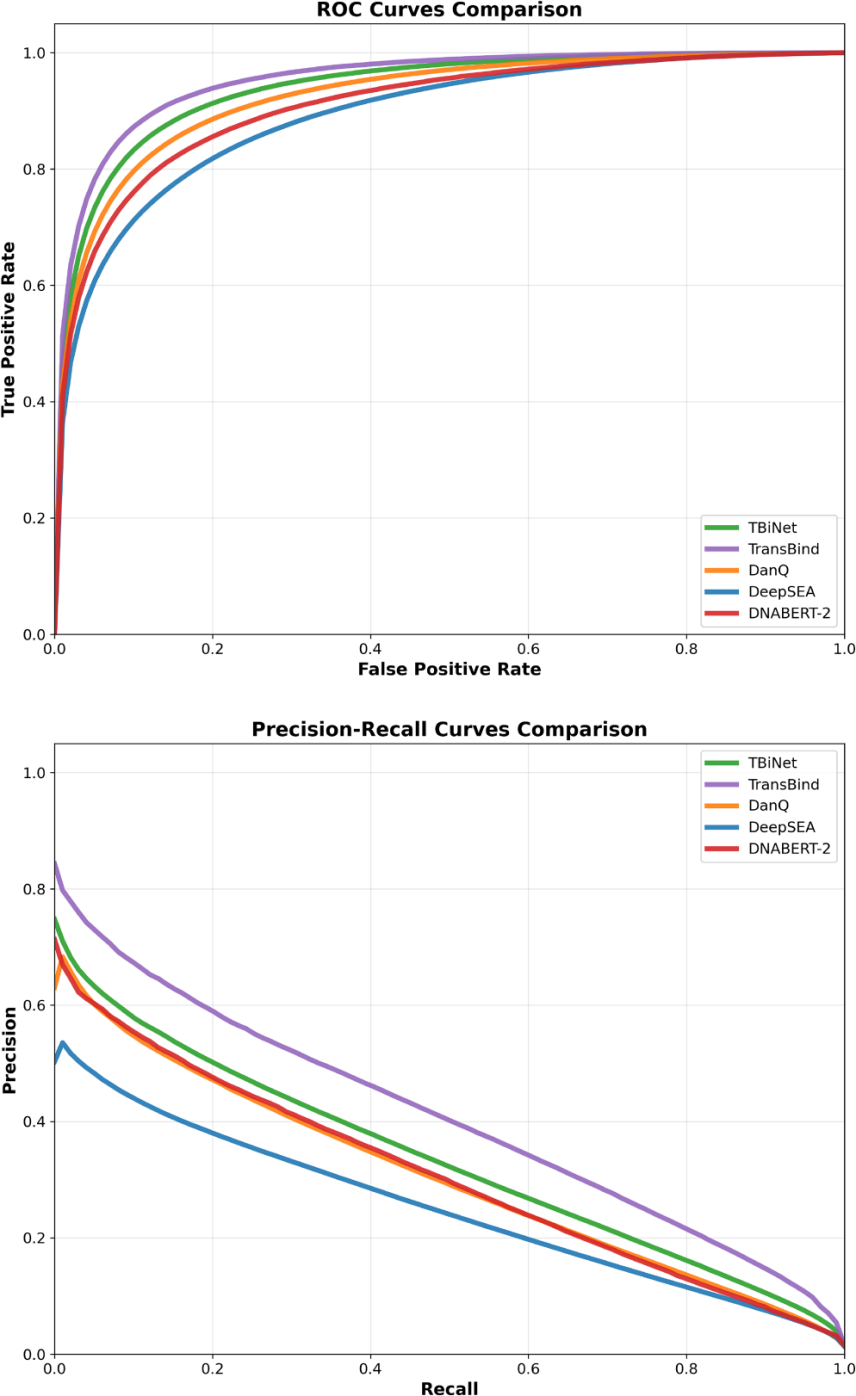
Average ROC curves (top) and precision–recall curves (bottom) across all TF-cell type combinations for all methods. TransBind demonstrates superior performance in both metrics, with particularly notable improvements in the precision–recall space.

Figure 3 presents the average ROC and PR curves across all 690 TF-cell type combinations across all methods. The ROC curves show TransBind’s ability to achieve higher true positive rates while maintaining lower false positive rates compared to baseline methods. The PRC curves indicate TransBind obtains higher precision and recall across board.

**Figure 4.**
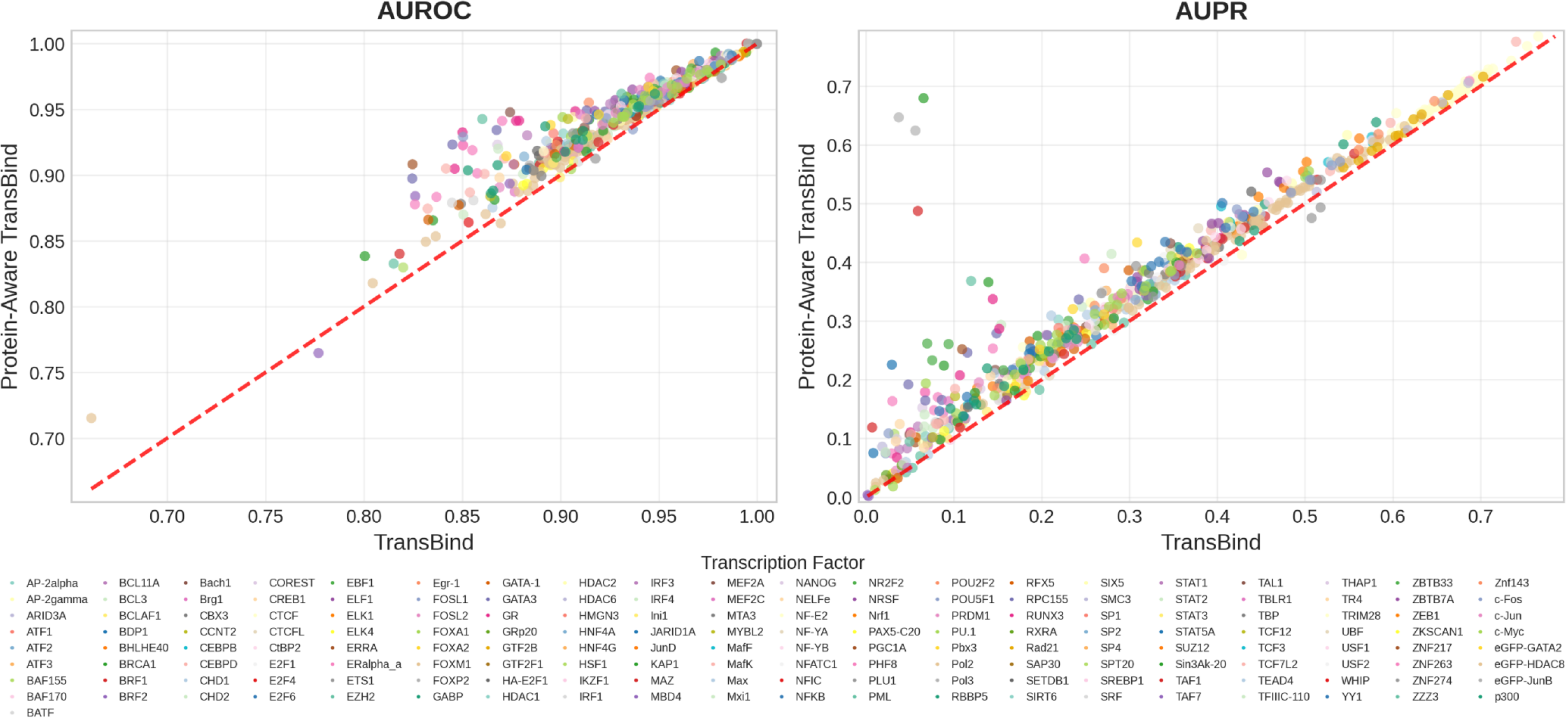
Performance comparison of TransBind versus TBiNet across 690 TF-cell type combinations involving 161 TFs and 91 cell types in the test set. Each point represents a TF-cell type experiment in terms of AUROC (left) and AUPR (right). Points above the diagonal line indicate better performance by TransBind. Each color denotes each unique TF.

To assess the performance across individual TFs, we conducted a fine-grained comparison between TransBind and TBiNet across all 690 TF–cell type combinations in the test dataset (Fig. 4). TransBind outperformed TBiNet in 97.1% (670/690) of cases in terms of AUROC and 98.0% (676/690) in terms of AUPR, underscoring its robustness and broad applicability. The individual performance curves for each TF-cell type experiment are provided in Supplementary Figs S2 and S3.

The improvement is especially meaningful in biological contexts where the underlying data is highly imbalanced and the cost of false positives is high.For example, in many challenging cases where TBiNet failed, TransBind achieved dramatic gains, such as Pol3 in K562 (AUPR 0.037 → 0.647) and BRCA1 in K562 (0.066 → 0.679). Even in many easier cases where the baseline performance was already good, TransBind still yielded notable improvements, e.g. CTCF in H1-hESC (0.604 → 0.654). Higher AUPR values translate into more precise identification of true binding sites, enabling more efficient downstream experimental validation.

### Convolutional kernels capture general DNA patterns

To evaluate whether TransBind captures genuine regulatory mechanisms that are biologically explainable, we systematically analyzed the learned convolutional filters with respect to TF binding motifs. We extracted the trained weight matrices of all 320 kernels in the first CNN layer in TransBind and queried them against the known TF binding motifs in the JASPAR [30] database using TOMTOM [31] similarity analysis.

TransBind achieved excellent motif discovery performance, learning 160 biologically meaningful kernels matching known TF binding sites in JASPAR with statistical significance ($P < .05$). Twenty-seven kernels reached highly significant matches ($P < 10^{-6}$), with the strongest discovery achieving $P = 1.29 \times 10^{-8}$ for the glucocorticoid response element binding protein Gmeb1.

The discovered motifs encompass diverse regulatory pathways essential for cellular function. The most prevalent binding motifs belong to CTCF (36 kernels), the master organizer of chromatin architecture and genomic looping. Additional discoveries span critical regulatory programs, such as developmental patterning (Six4, Lhx1), immune response (Nfatc2), metabolic control (HNF1A), and chromatin remodeling factors. The breadth of the discovery demonstrates that TransBind learned fundamental principles of gene regulation across multiple biological processes.

**Figure 5.**
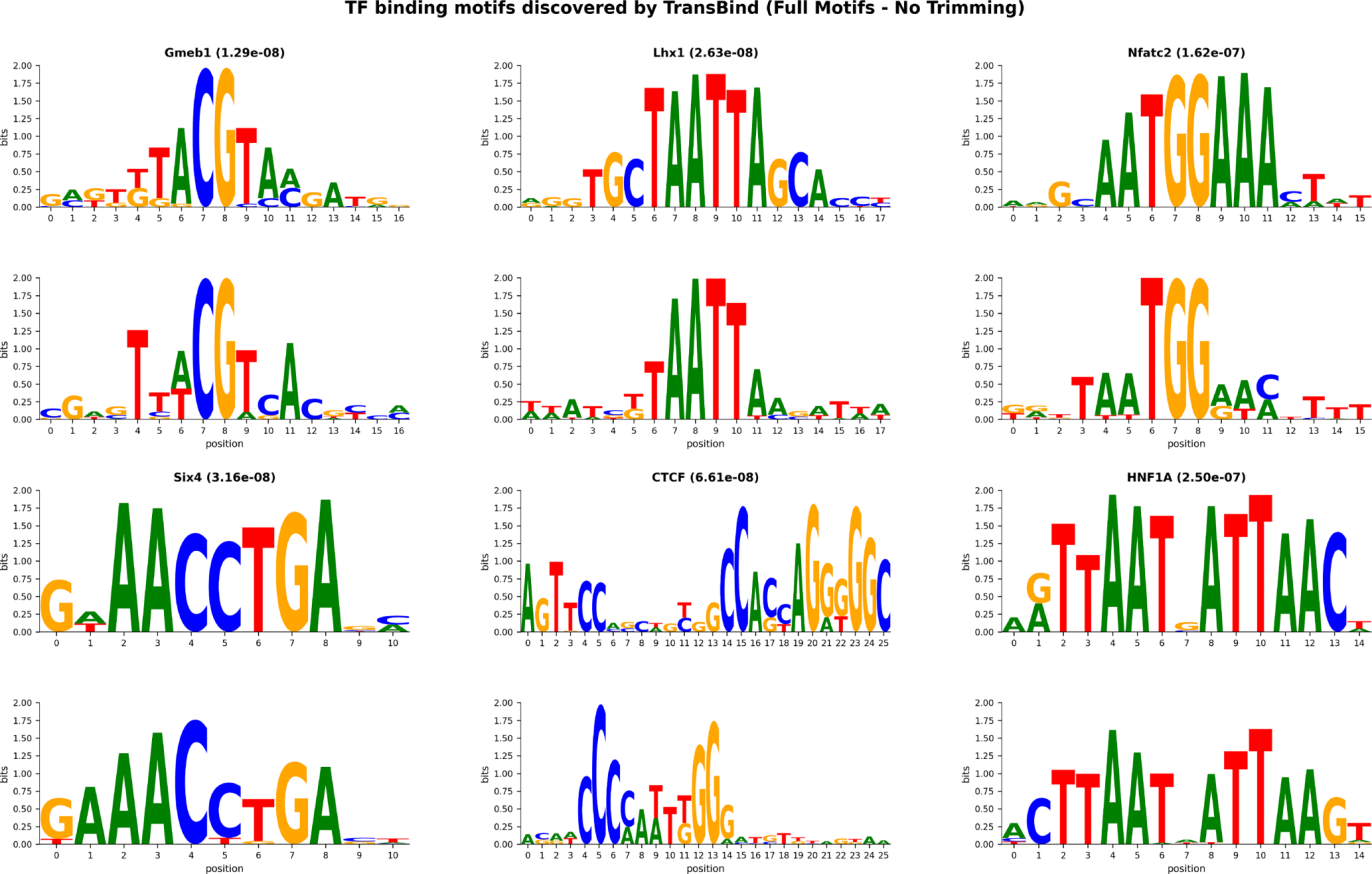
TF binding motifs discovered by TransBind. Each pair of logos for a TF shows the JASPAR reference motif (top) compared with the corresponding motif learned by TransBind (bottom). TF names and statistical significance (*P*-values) are shown above each pair. All discoveries have $P < 2 \times 10^{-7}$, demonstrating that TransBind successfully learned biologically meaningful binding patterns for diverse TF families including chromatin organizers (CTCF), homeobox factors (Six4, Lhx1), immune regulators (Nfatc2), and metabolic factors (HNF1A, Gmeb1).

Fig. 5 presents sequence logo comparisons between TransBind’s top motif discoveries and their JASPAR references. The striking visual correspondence—particularly for Six4, Gmeb1, and HNF1A—provides compelling evidence that TransBind captured authentic regulatory grammar rather than dataset-specific patterns. Importantly, these motifs were learned *de novo* from sequence data alone, without explicit knowledge of binding site locations.

### TF-conditioned motif validation

While convolutional kernels capture general DNA patterns, TF-specific motif recognition emerges through integration of protein embeddings via cross-attention. To validate this, we performed attribution analysis using Integrated Gradients [32] conditioned on individual TFs. For each TF, we computed position-specific attribution scores with respect to the TF’s output while conditioning on its protein embedding, extracted high-attribution regions, constructed position weight matrices, and validated against JASPAR 2024 using TOMTOM ($P < 0.01$ significance threshold).

We validated this TF-specific learning on nine representative TFs spanning seven structural families (Table 3). For the zinc-finger superfamily, GATA1 yielded ATTGTAATT matching JASPAR MA0140.3 (GATA3, $P = 5.4 \times 10^{-7}$), CTCF extracted its characteristic multiblock binding site (MA1929.2, $P = 2.0 \times 10^{-6}$), and SP4 recovered the GC-rich CGGGGCGGGG motif (MA0685.2, $P = 1.7 \times 10^{-5}$). For the bZIP family, both MAFK and JUND extracted TGACTCA, matching the MARE (MA0496.4, $P = 3.7 \times 10^{-5}$) and TRE (MA0491.3, $P = 9.5 \times 10^{-5}$), respectively, demonstrating subfamily-specific recognition within shared sequence preferences. Additional families validated include bHLH (MYC: CACGTG E-box, MA0059.2, $P = 3.5 \times 10^{-5}$), Forkhead (FOXA1: TGTTTAC, MA0148.5, $P = 9.0 \times 10^{-5}$), E2F (E2F4: TTTCGCGCCTC, MA0470.3, $P = 2.7 \times 10^{-5}$), and STAT (STAT3: TTCGAAGAA, MA0144.3, $P = 5.4 \times 10^{-5}$).

**Table 3.** Representative examples of TF-conditioned attribution analysis demonstrating TF-specific motif recognition

TF	Family	Consensus	JASPAR match	JASPAR ID	*P*-value
GATA1	Zinc-finger (GATA)	ATTGTAATT	GATA3	MA0140.3	$5.4 \times 10^{-7}$
CTCF	Zinc-finger (CTCF)	GGCGGAGGACCCCCG	CTCF	MA1929.2	$2.0 \times 10^{-6}$
SP4	Zinc-finger (SP/KLF)	CGGGGCGGGG	SP4	MA0685.2	$1.7 \times 10^{-5}$
E2F4	E2F	TTTCGCGCCTC	E2F4	MA0470.3	$2.7 \times 10^{-5}$
MYC	bHLH	CACGTG	MYC	MA0059.2	$3.5 \times 10^{-5}$
MAFK	bZIP-MARE	TGACTCA	MAFK	MA0496.4	$3.7 \times 10^{-5}$
STAT3	STAT	TTCGAAGAA	STAT3	MA0144.3	$5.4 \times 10^{-5}$
FOXA1	Forkhead	TGTTTAC	FOXA1	MA0148.5	$9.0 \times 10^{-5}$
JUND	bZIP-AP1	TGACTCA	JUND	MA0491.3	$9.5 \times 10^{-5}$

These representative examples demonstrated high statistical significance ($P < 10^{-4}$), confirming that integration of TF protein embeddings via cross-attention enables recognition of cognate binding motifs across structurally diverse TF families.

### Label-zero-shot binding prediction for TF paralogs

To rigorously evaluate label-zero-shot generalization to TF paralogs, we performed leave-three-out cross-validation (LOTO-CV) across 161 TFs using 8 independent folds. Each fold trained a separate model on 158 TFs and evaluated on a held-out set of 3 TFs, which were excluded from all training and validation splits. Across all folds, this evaluation scheme covered 24 TFs spanning 15 distinct families (Table 4), ensuring comprehensive assessment across diverse DNA-binding domains and regulatory roles.

**Table 4.** Leave-three-out cross-validation label-zero-shot generalization performance across 24 held-out TFs

TF	AUPR	AUROC
FOXA1	0.3466	0.7220
GABP	0.5463	0.8951
STAT1	0.1670	0.7586
ATF2	0.2993	0.5064
ELF1	0.5207	0.7539
ETS1	0.2621	0.7077
FOXP2	0.3309	0.7317
CEBPB	0.2038	0.6877
c-FOS	0.3013	0.7181
c-JUN	0.2256	0.8187
FOXM1	0.5564	0.7161
GATA2	0.3696	0.8051
GATA3	0.1999	0.5363
STAT3	0.2105	0.5412
STAT5A	0.1483	0.5813
NFKB	0.2678	0.7677
c-Myc	0.2362	0.7331
SP1	0.2314	0.6757
Max	0.3560	0.7574
GR	0.1865	0.5837
HNF4A	0.5742	0.8693
BAF155	0.2575	0.8072
CTCF	0.4015	0.7328
IRF1	0.2150	0.7811
Overall (24 TFs)	$0.3089 \pm 0.128$	$0.7162 \pm 0.102$

The 161-TF dataset exhibits substantial sequence diversity across families, with mean pairwise identity of 19.13% (median: 20.00%, range: 3.77%–77.33%). Only one TF pair exceeded 70% sequence identity (HDAC1/HDAC2: 77.33%), confirming broad coverage of evolutionarily divergent TF families and validating the stringency of the LOTO-CV protocol for assessing true label-zero-shot generalization.

TransBind achieves strong label-zero-shot generalization performance, with a macro-averaged AUROC of $0.7162 \pm 0.102$ and macro-averaged AUPR of $0.3089 \pm 0.128$ across the 24 held-out TFs. Critically, performance remains stable across TF families, with no TF exhibiting near-random behavior (minimum AUROC: 0.506 for ATF2), demonstrating that the model successfully captures generalizable binding patterns despite never encountering these TFs during training. Given the severe class imbalance inherent to TF binding prediction, these results represent robust generalization to unseen TF paralogs and validate the protein-aware architecture’s capacity to transfer binding knowledge across the TF sequence space.

Characterization of paralog relationships. To quantify the degree of sequence similarity between held-out and training TFs, we computed global sequence identity for each held-out TF against all training TFs. Held-out TFs exhibit a mean identity of 45.86% to their closest training paralog (range: 35.33%–67.51%). Seven TFs (29%) have paralogs exceeding 50% identity, including HNF4A (closest to HNF4G: 67.5%), FOXA1 (closest to FOXA2: 65.9%), and c-JUN (closest to JunD: 61.4%). The remaining 17 TFs (71%) show more distant relationships (less than 50% identity), with several having no close paralogs above 40% identity. Consequently, this evaluation primarily demonstrates within-family generalization the ability to predict binding for TF paralogs by leveraging family-level binding preferences learned from related training TFs rather than cross-family generalization to entirely novel TF families.

We emphasize that while held-out TFs are completely excluded from the supervised training on TF–DNA binding labels in this project, their protein sequences may have been included in the pretraining corpus of the PLM ESM–DBP. Consequently, this constitutes a label-zero-shot setting rather than a strict sequence-zero-shot setting, in which: the protein encoder may have been exposed to the held-out TF sequences during unsupervised pretraining, but the TransBind model has never observed TF–DNA binding labels for these TFs.

### Protein sequence information enables label-zero-shot generalization

To isolate the contribution of protein sequence information to label-zero-shot performance, we compared TransBind_zeroshot against a strong DNA sequence-only baseline specifically adapted for the label-zero-shot pairwise prediction task. This controlled comparison directly tests whether label-zero-shot gains arise from incorporating protein sequence priors versus merely reformulating the prediction objective or conditioning on TF identity.

We adapted TBiNet into a TF-ID conditioned model that takes DNA sequences and a TF identifier embedding as input, enabling it to make predictions for held-out TFs in the same pairwise manner as TransBind_label_zeroshot. Critically, this baseline uses the identical training procedure, hyperparameters, and evaluation protocol as TransBind, differing only in the absence of protein sequence information. This ensures that any performance differences reflect the value of protein priors rather than architectural or optimization advantages.

We evaluated both models on three TFs—FOXA3, ATF4, and HNF1A—that were entirely excluded from the 161-TF training set. These three TFs were selected to span distinct DNA-binding domain families and include both moderately and distantly related paralogs, providing a representative label-zero-shot evaluation. These TFs exhibit moderate sequence similarity to TFs in the training set: ATF4 (38.2% identity to JunB), HNF1A (38.7% identity to ELF1), and FOXA3 (50.4% identity to FOXA1), with a mean of 42.4% (range: 38.2%–50.4%). Two TFs (ATF4, HNF1A) show particularly distant relationships ($< 40\%$ identity to any training TF), while FOXA3 has two paralogs (FOXA1, FOXA2) exceeding 50% identity.

As shown in Table 5, TransBind consistently outperforms the TF-ID conditioned TBiNet baseline across all three TFs in both AUROC and AUPR. For example, on FOXA3, TransBind achieves an AUROC of 0.603 compared to 0.545 for the DNA sequence-only baseline (gain: 5.8 percentage points), and an AUPR of 0.577 compared to 0.528 (gain: 4.9 percentage points). Similar improvements are observed for ATF4 and HNF1A.

**Table 5.** Comparison of TransBind_label_zeroshot with a TF–ID conditioned DNA sequence-only baseline (TBiNet) under a label-zero-shot setting

TF	TransBind_zeroshot		TF-ID conditioned TBiNet	
	AUROC	AUPR	AUROC	AUPR
FOXA3	0.6031	0.5768	0.5454	0.5283
ATF4	0.5750	0.5356	0.5475	0.5260
HNF1A	0.6287	0.5990	0.5667	0.5418

The consistent improvements across all three TFs (AUROC gains: 2.7–6.2 percentage points; AUPR gains: 0.96–4.9 percentage points) demonstrate that protein priors enable meaningful generalization.

## Ablation study

We systematically evaluate the contribution of each component in TransBind. All ablation experiments use identical training procedures, optimization settings, and evaluation protocols, differing only in the component under investigation.

### Transcription factor information and representations

Table 6 compares models with different protein feature representations. The DNA sequence-only baseline achieves 0.9488 AUROC and 0.3634 AUPR, suggesting that the DNA encoder captures key binding motifs. Adding protein information consistently improves performance across all variants. The hand-crafted features (protein sequence length) provide modest gains (+0.0007 AUROC, +0.0049 AUPR), while the learned TF embeddings (the TF features learned by neural networks from the training data without using pretrained PLMs) slightly improve performance (0.9498 AUROC, 0.3686 AUPR).

**Table 6.** Effect of TF information and representations

TF Representation	AUROC	AUPR	Parameters
Sequence only	0.9488	0.3634	4.4M
Hand-crafted features	0.9495	0.3683	4.5M
Learned TF embedding	0.9498	0.3686	4.5M
ESM protein embedding	0.9503	0.3701	4.6M
**ESM–DBP (TransBind)**	**0.9508**	**0.3741**	4.6M

In contrast, the Pretrained PLM embeddings substantially improve performance. The ESM embeddings alone already outperform the learned and hand-crafted features, while the ESM–DBP embeddings used in TransBind achieve the best results. This suggests that pretraining on DNA-binding proteins provides additional task-relevant inductive bias beyond generic protein representations.

### DNA sequence encoder components

Table 7 shows the contribution of BiLSTM and Transformer components. The full BiLSTM + Transformer model achieves 0.9508 AUROC and 0.3741 AUPR. Removing either component degrades performance: Transformer-only achieves 0.9428 AUROC and 0.3536 AUPR, while BiLSTM-only achieves 0.9418 AUROC and 0.3435 AUPR. This indicates that the BiLSTM captures more local sequence dependencies, while the Transformer models more long-range interactions.

**Table 7.** Effect of DNA sequence encoder components

Model variant	AUROC	AUPR	Parameters
Transformer only	0.9428	0.3536	3.4M
BiLSTM only	0.9418	0.3435	3.6M
**TransBind**	**0.9508**	**0.3741**	4.6M

### Multimodal fusion strategies

We compare concatenation, bidirectional cross-attention, and unidirectional cross-attention (TransBind) for combining DNA and protein features (Table 8).

**Table 8.** Effect of multimodal fusion strategies

Fusion strategy	AUROC	AUPR	Parameters
Concatenation	0.9498	0.3682	4.1M
Bidirectional cross-attention	0.9499	0.3688	5.3M
**TransBind**	**0.9508**	**0.3741**	4.6M

Concatenation achieves 0.9498 AUROC and 0.3682 AUPR with 4.1M parameters. Bidirectional cross-attention increases parameters to 5.3M but slightly increases AUPR (0.3688). Unidirectional cross-attention, where DNA features query protein features, achieves the best performance (0.9508 AUROC, 0.3741 AUPR) with 4.6M parameters, reflecting the biological intuition that protein structure plays an important role in determining DNA-binding specificity.

### Effect of input DNA sequence length on model performance

To assess the impact of input DNA sequence length, we evaluated model performance using DNA sequences of length 500, 800, 1000, and 1200 bp (Table 9). Performance consistently improves from 500 bp (AUROC 0.9422, AUPR 0.3524) to 800 bp (AUROC 0.9482, AUPR 0.3643 ), and reaches its maximum at 1000 bp (AUROC 0.9508, AUPR 0.3741 ).

**Table 9.** Effect of input sequence length on model performance

Sequence length (bp)	AUROC	AUPR
500	0.9422	0.3524
800	0.9482	0.3643
**1000**	**0.9508**	**0.3741**
1200	0.9501	0.3710

Increasing the sequence length to 1200 bp results in a slight decrease in performance (AUROC 0.9501, AUPR 0.3710), suggesting that additional flanking regions beyond 1000 bp provide diminishing returns. Therefore, we use 1000 bp as the default input length in all subsequent experiments.

Overall, the ablation results demonstrate that TransBind’s performance gains arise from the complementary integration of protein representations, hierarchical DNA sequence modeling, and biologically motivated multimodal fusion, rather than from any single component alone.

## Discussion

This study addresses a key gap in genome-wide TF–DNA binding prediction: almost all existing methods ignore TF sequence and structural information, despite its critical role in determining binding specificity. By integrating TF embeddings generated by PLMs with DNA sequence features through the cross-attention mechanism, TransBind enables TF-specific scanning of genomic sequences. This protein-aware framework delivers consistent and substantial gains in prediction accuracy over state-of-the-art deep learning methods. Significantly, it performs better in 98% of the 690 TF–cell type combinations tested than the second most accurate method—TBiNet. Beyond accuracy, TransBind recovers 160 known motifs of diverse TF families, demonstrating the biological interpretability of the model and the ability to learn regulatory patterns from scratch.

Moreover, due to the use of protein embeddings of TFs as input, TransBind_zeroshot also exhibits label-zero-shot generalization to unseen TFs, offering predictive capability for TFs with no available binding data for training, a critical step toward the scalable annotation of many uncharacterized TFs.

These improved or new capabilities have practical implications for regulatory genomics, including prioritizing TFs for experimental validation, designing synthetic regulatory sequences, and aiding variant interpretation in phenotype-associated non-coding regulatory regions in genomes.

However, the approach still has some limitations. The performance of the label-zero-shot prediction (i.e. AUROC between 0.575 and 0.628) is moderate, highlighting the challenge of modeling complex protein–DNA recognition without TF-specific training examples. The relatively lower performance may be largely due to the limited amount of training data of only 161 TFs. To further improve its generalization performance, a much larger training dataset involving hundreds or more TFs may be needed. In addition, the input for the label-zero-shot prediction in TransBind_zeroshot does not implicitly contain cell type information as the multilabel classifier TransBind does, which may also lead to lower prediction accuracy.

Indeed, the current architecture of TransBind predicts TF–DNA binding from TF and DNA information only, without considering the accessibility of chrommatin and the higher-order chromatin architecture that often influences in vivo binding patterns. Moreover, protein embeddings derived from the PLM contain only protein sequence and indirect structural information, without directly leveraging the 3D structures of TFs predicted by deep learning methods[33, 34]. Addressing these limitations will require integrating additional sources of data such as ATAC-seq chromosome accessibility data, Hi-C chromosome conformation data, predicted structures of TFs, genome annotations, genome/histone methylation, and gene expression data. It is worth noting that dynamic chromosome accessibility and gene expression data can not only enable the method to predict biological context-specific TF–DNA binding but also provide implicit cell type information for predicting cell type specific TF–DNA binding. By situating TF binding prediction within a multimodal framework leveraging multiple sources of omics and protein structure data, more generalizable and biologically grounded models of TF–DNA binding prediction can be developed.

Finally, TransBind was currently trained on the human TF–DNA binding data. However, we image it can be trained on the TF–DNA binding data of multiple species because it can learn the general biophysical interactions between TFs and DNAs that are assumed to be universal across species. In the future, we plan to curate a large dataset consisting of TF–DNA binding data of many species and train and test TransBind on it. We will test if TransBind can generalize to TFs in new species that are not used in training at all.

## Supplementary Material

lqag047_Supplemental_File

## Data Availability

TransBind is an open-source software package, and its source code is publicly available via Zenodo at https://doi.org/10.5281/zenodo.19462292.
