# Supplementary material for: Integrating protein and DNA embeddings for improving genome-wide transcription factor binding site prediction
Source: NAR Genom Bioinform. 2026 May 6;8(2):lqag047. doi: 10.1093/nargab/lqag047 (PMC13145115; doi:10.1093/nargab/lqag047)
Supplement: lqag047_Supplemental_File [file lqag047_supplemental_file.pdf]

Published online *Nucleic Acids Research, 2025, Vol. 00, No. 00* **1–5**
doi:10.1093/nar/gkn000

Supplementary Materials: Integrating Protein and DNA Embeddings for Improving Genome-Wide Transcription Factor Binding Site Prediction

Shreya Basnet[1] **and Jianlin Cheng**[1,*]

[1]Department of Electrical Engineering & Computer Science, University of Missouri, Columbia, MO 65211, USA
[*]Corresponding author: chengji@missouri.edu

HYPERPARAMETER OPTIMIZATION AND TRAINING CONFIGURATION

Overview

To optimize model performance, we conducted comprehensive hyperparameter tuning using the Optuna framework with Tree-structured Parzen Estimator (TPE) sampling and MedianPruner for efficient early stopping. The optimization targeted key architectural and training parameters to maximize validation set performance as measured by AUPR and AUROC.

Hyperparameter Search Space

The following hyperparameters were explored during optimization:

- **Learning rate:** Log-uniform distribution in $[5\times10^{-5}, 5\times10^{-3}]$
- **Dropout probability:** Uniform distribution in $[0.05, 0.4]$
- **Weight decay:** Log-uniform distribution in $[1\times10^{-4}, 1\times10^{-1}]$
- **CNN output channels:** Categorical choice from $\{256, 320, 384, 512\}$
- **CNN kernel size:** Categorical choice from $\{19, 26, 35, 45\}$
- **LSTM hidden size:** Categorical choice from $\{128, 160, 192, 256\}$
- **LSTM layers:** Integer range $[1, 3]$
- **Attention heads:** Categorical choice from $\{4, 8, 16\}$
- **Feedforward dimension:** Categorical choice from $\{512, 1024, 1280, 2048\}$
- **FC1 size:** Categorical choice from $\{512, 695, 1024, 1536\}$

Optimal Configuration

After systematic hyperparameter search, the following optimal configuration was selected based on validation performance:

- Learning rate: 3.28×10^{-4}
- Weight decay: 0.028
- Dropout: 0.088
- CNN output channels: 320
- CNN kernel size: 26
- LSTM hidden size: 160
- LSTM layers: 2
- Attention heads: 16
- Feedforward dimension: 1024
- FC1 size: 1024

This configuration achieved optimal balance between model capacity and generalization performance on the validation dataset.

© The Author(s)
This is an Open Access article distributed under the terms of the Creative Commons Attribution Non-Commercial License (http://creativecommons.org/licenses/by-nc/2.0/uk/) which permits unrestricted non-commercial use, distribution, and reproduction in any medium, provided the original work is properly cited.

Table S1. Summary of hyperparameter optimization for baseline models.

Model	Learning Rate	Dropout	Weight Decay	CNN/Conv1D Channels	CNN Kernel Size
DeepSEA	0.01	0.20	1e–08	320	26
DanQ	0.001	0.20	1e–08	320	26
TBiNet	0.001	0.20	1e–08	320	26
DNABERT-2	1.216e–5	0.0195	1.25e–4	–	–

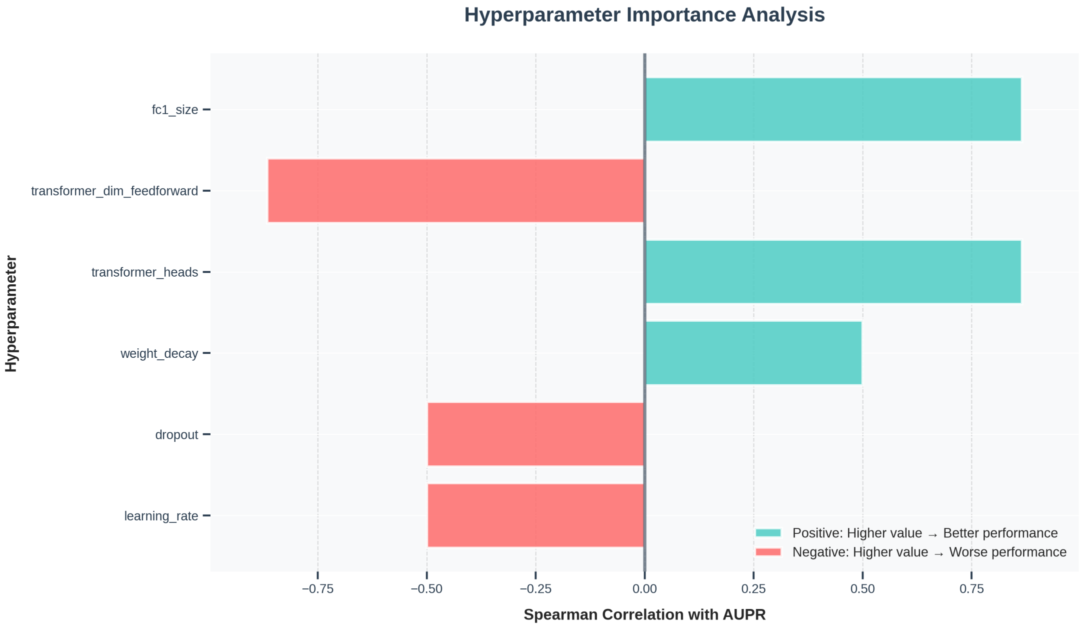

Figure S1. Spearman correlation-based sensitivity analysis for key hyperparameters. Each bar indicates the strength and direction of the monotonic relationship between the hyperparameter value and validation AUPR, demonstrating which hyperparameters most strongly influence model performance.

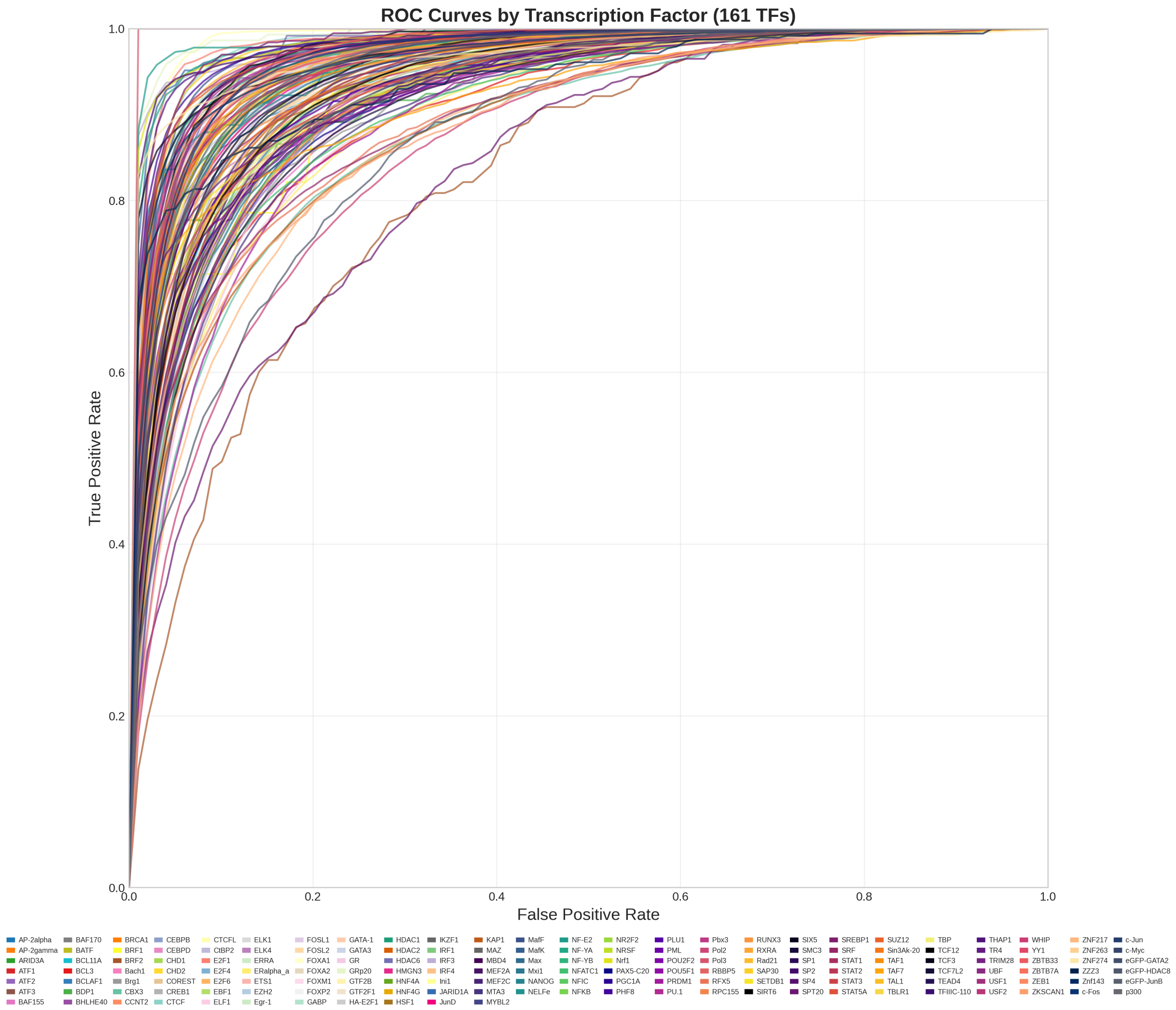

Figure S2. ROC curves for individual transcription factors in the test dataset. Each curve (colored distinctly) represents the performance for one unique TF, demonstrating the model's ability to discriminate DNA-binding sites across diverse transcription factor families.

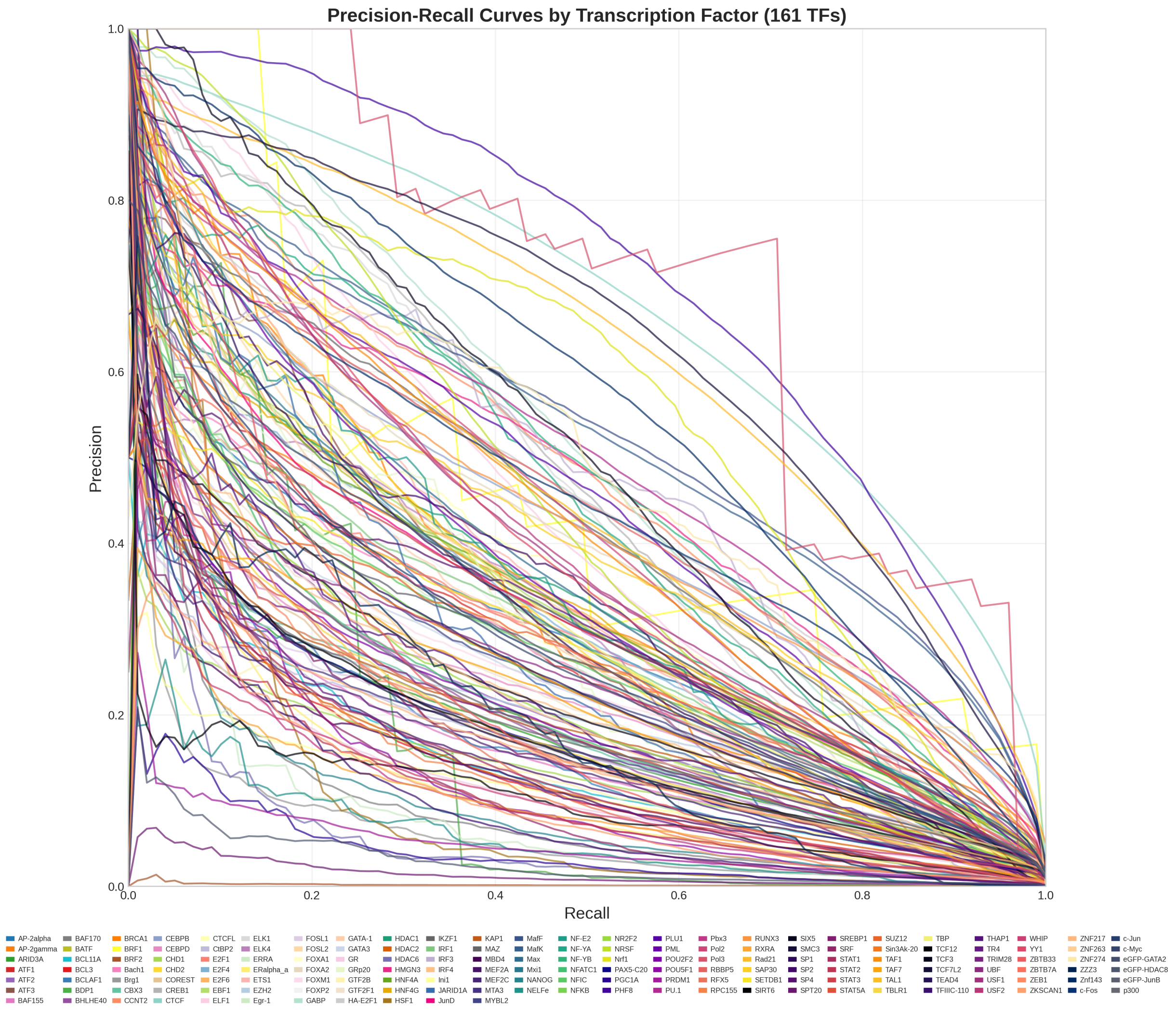

Figure S3. Precision-recall curves for individual transcription factors in the test dataset. Each curve (colored distinctly) represents one unique TF, illustrating the model's precision-recall trade-off across different transcription factor binding profiles.
